# Undiagnosed *Cryptococcus gattii* meningitis leading to subsequent ventriculoperitoneal shunt infection in a patient with symptoms of normal pressure hydrocephalus: case report and literature review

**DOI:** 10.1186/s12879-018-3165-y

**Published:** 2018-06-04

**Authors:** Wutthiseth Dhitinanmuang, Piriyaporn Chongtrakool, Anupop Jitmuang

**Affiliations:** 1 Division of Infectious Diseases and Tropical Medicine, Department of Medicine, Faculty of Medicine Siriraj Hospital, Mahidol University, 2 Wanglang Road, Bangkoknoi, Bangkok, 10700 Thailand; 2 Department of Microbiology, Faculty of Medicine Siriraj Hospital, Mahidol University, Bangkok, Thailand

**Keywords:** *Cryptococcus gattii*, Cryptococcal meningitis, Ventriculoperitoneal shunt infection, Infected ventriculoperitoneal shunt pseudocyst

## Abstract

**Background:**

*Cryptococcus gattii* is known to be an etiologic agent of human cryptococcosis, particularly in immunocompetent persons. *C. gattii* infection usually involves the central nervous system, the respiratory tract, or may be disseminated. Here we report an atypical manifestation of *C. gattii* infection in a patient who had *C. gattii* meningitis complicating the ventriculoperitoneal (VP) shunt infection and concurrent infected intraabdominal VP shunt pseudocyst.

**Case presentation:**

A 66-year-old Thai female was initially diagnosed with normal pressure hydrocephalus (NPH) and underwent programmable VP shunt placement. However, she still suffered from recurrent communicating hydrocephalus with in-place VP shunt, and later developed recurrent gait impairment, chronic abdominal pain and abdominal mass. Radiological studies demonstrated recurrent hydrocephalus and a very large intraabdominal VP shunt pseudocyst. *C. gattii* was isolated from both the cerebrospinal fluid and the pseudocyst aspiration. *C. gattii* meningitis complicating the VP shunt infection and concurrent infected intraabdominal VP shunt pseudocyst was diagnosed. Prolonged antifungal therapy, removal of the infected VP shunt with subsequent implant of a new shunt provided a good outcome.

**Conclusion:**

Chronic *C. gattii* meningitis should be aware in a patient presenting with normal pressure hydrocephalus. Under-diagnosed cryptococcal meningitis following VP shunt insertion for treating the hydrocephalus can render a complicated VP shunt infection including infected VP shunt pseudocyst.

## Background

Cryptococcosis is one of the more common systemic fungal infections caused by two main species, *Cryptococcus neoformans* and *Cryptococcus gattii*. *Cryptococcus* is a spore forming, environmental encapsulated fungus [1, 2]. Formerly, human *Cryptococcus* had been grouped into 4 major serotypes based on antigenic differences of the polysaccharide capsules, namely serotypes A, B, C and D [1, 3]. *C. neoformans* was categorized into serotypes A and D whereas *C. gattii* was classified into the serotypes B and C. Inhalation of spores is a primary route of infection that mainly affects immunocompromised persons, such as HIV-infected patients, organ transplant recipients, and patients receiving corticosteroid or immunosuppressive agents. However, several published reports have demonstrated *C. gattii* to be frequently associated with cryptococcosis in patients with no known immunodeficiency [4–6].

In recent years, the prevalence of *C. gattii* infection has increased since a major outbreak on Vancouver Island [4]. Similar to *C. neoformans,* central nervous system (CNS) and respiratory tract infections are the most common presentations [1–4]. However, *C. gattii* infection occurs more frequently in immunocompetent persons and more often results in CNS complications, such as hydrocephalus, and large cryptococcoma with delayed treatment response [1, 2, 7]. Few studies of *C. gattii* infection in Thailand have been published. A molecular typing study of 498 *Cryptococcus* spp. isolates from clinical, animal and environmental sources from Thailand found only 13 isolates (2.6%) were *C. gattii*, but clinical data have not been reported [3]. Another study described Tsunami survivors from Thailand who suffered from primary cutaneous cryptococcosis caused by *C. gattii* [5]. Here we report atypical *C. gattii* infection in a patient who had chronic *C. gattii* meningitis complicating a ventriculoperitoneal (VP) shunt infection and concurrent infected intraabdominal VP shunt pseudocyst.

## Case presentation

A 66-year-old Thai female with underlying hypertension was admitted to Siriraj Hospital, Bangkok, Thailand on September 26, 2016 to evaluate the function of an implanted VP shunt and identify the cause of an abdominal mass. Two years earlier, she had progressive memory impairment, dizziness, and difficulty walking for 4 months. Computed tomography (CT) of the brain revealed communicating hydrocephalus without leptomeningeal enhancement or abnormal enhancing lesion. Initial cerebrospinal fluid (CSF) showed a white blood cell (WBC) count of 54 cells/mm^3^ with 96% lymphocytes, a protein 165 mg/dL, and a ratio of CSF/serum glucose of 25/126 mg/dL (0.2). The patient was diagnosed with normal pressure hydrocephalus (NPH) and underwent programmable VP shunt placement to relieve her symptoms. One year later, the gait difficulty and dizziness resumed. A brain CT showed recurrent communicating hydrocephalus. The neurological deficits were reduced after adjusting the VP shunt pressure. Six months following adjustment of the shunt pressure, the patient developed abdominal discomfort with a palpable mass at the right paraumbilical region. The patient did not feel feverish and did not have nausea or vomiting. Prior to this admission, gait difficulty and dizziness including diffuse headache recurred. Recurrent hydrocephalus was suspected and the patient was admitted for further investigation.

**Fig. 1:**
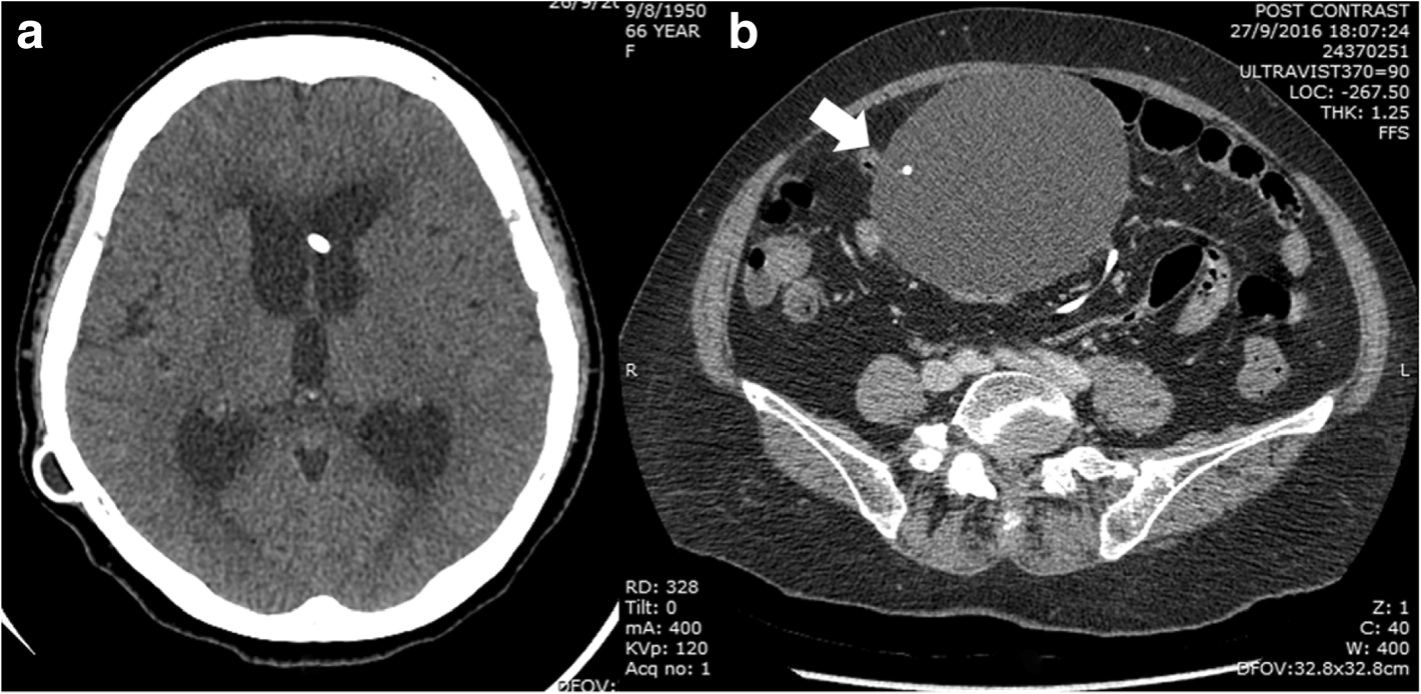
a) Computed tomography of brain revealed recurrent communicating hydrocephalus with appropriate in-place VP shunt, b) Computed tomography of whole abdomen with contrast revealed a large well-defined cystic mass at inframesocolic space, approximately 9.6 × 11.3 × 15 cm in diameter which it encased the distal limb of VP shunt (arrow)

The patient was afebrile and had a very large, ill-defined mass with cystic consistency and smooth surface at the right paraumbilical region. Neurological examination revealed a magnetic gait, no neck stiffness and otherwise normal findings. The brain CT revealed communicating hydrocephalus with appropriate in-place VP shunt as shown in Fig. 1a. The abdominal CT identified a well-defined rounded cystic mass measuring approximately 9.6 × 11.3 × 15 cm at the inframesocolic space. This cystic mass encased the distal end of the VP shunt, suggestive of a CSF pseudocyst (Fig. 1b)**.** CSF was collected by lumbar puncture and returned a WBC count of 12 cells/mm^3^ with 94% lymphocytes, 20 mg/dL protein, and a ratio of CSF/serum glucose of 46/106 mg/dL (0.43). Ultrasonography-guided aspiration of the intraabdominal VP shunt pseudocyst was also performed. A total of 360 mL of clear yellow fluid was obtained and examined. The WBC count was 20 cell/mm^3^ with 97% lymphocytes, 472 mg/dL protein, and a ratio of CSF/serum glucose of < 4.32/120 mg/dL (< 0.03). An India ink preparation of the CSF taken from the pseudocyst and from the lumbar puncture revealed a few encapsulated budding yeasts. Cryptococcal antigen testing of the serum and the CSF was positive at the titers of 1:8 and of > 1:1024, respectively. The yeasts grew well on saboraud dextrose agar without cycloheximide. The isolate was identified as *C. gattii* by biochemical testing and a conversion to blue color on L-canavanine-glycine bromothymol blue (CGB) agar. A molecular typing using the restriction fragment length polymorphism (RFLP) of *URA5* determined the isolate to be compatible with *C. gattii* molecular type VGI. Antifungal susceptibility testing by the broth microdilution method was performed and showed minimal inhibitory concentrations (MICs) of amphotericin B deoxycholate (ABD) of 0.5 μg/mL, 5-fluorocytosine (5-FC) of 0.5 μg/mL, fluconazole of 1 μg/mL, itraconazole of ≤ 0.015 μg/mL, voriconazole of 0.015 μg/mL, posaconazole of 0.03 μg/mL and all echinocandins (caspofungin, micafungin and anidulafungin) of > 8 μg/mL. Unfortunately, when NPH was initially diagnosed 2 yrs earlier the CSF culture had grown *C. gattii*, but the result was overlooked. Chest radiography found no pulmonary nodules or infiltrations. Complete blood count showed hemoglobin of 12.4 g/dL, hematocrit of 39.4%, WBC counts of 10,930/mm^3^ (neutrophil 70%, lymphocyte 25%, monocyte 5%) and platelet counts of 385,000/mm^3^. Blood urea nitrogen (BUN), creatinine, and liver function tests were within normal limits. Anti-HIV testing was negative. Immunological studies revealed CD_4_ T cell counts of 842 cells/mm^3^ (51.8%), CD_8_ T cell counts of 361 cells/mm^3^ (22.2%), IgG 1170 mg/dL, IgA 255 mg/dL and IgM 141 mg/dL. Anti-interferon gamma (anti-IFN-Ƴ) and anti-granulocyte-macrophage colony-stimulating factor (anti-GM-CSF) autoantibodies were both negative.

**Fig. 2:**
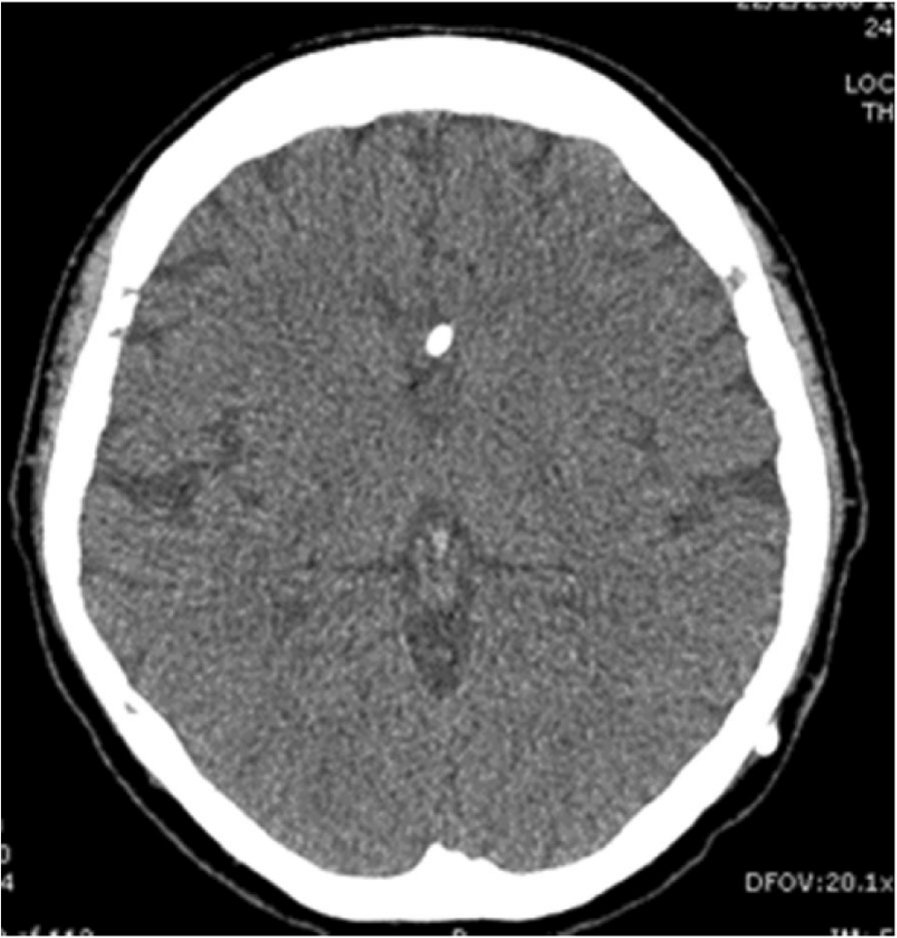
Computed tomography of brain following the shunt reimplantation exhibited significantly decreased ventricular dilatation

During hospitalization, the patient received daily intravenous amphotericin B deoxycholate 40 mg (0.7 mg/kg/day) combined with oral fluconazole 800 mg/day. The VP shunt was removed after 14 days of antifungal treatment. After discharge to home, the patient received long-term oral fluconazole for consolidation and maintenance therapy according to the IDSA recommendation [7]. Reimplantation of the VP shunt was performed after 8 weeks of antifungal treatment. The follow-up CSF culture was sterile. Brain CT following shunt reimplantation demonstrated significantly decreased ventricular dilatation (Fig. 2). After 6 months of antifungal treatment, the gait abnormality, dizziness and diffuse headache resolved, and the abdominal mass was no longer detectable.

## Discussion

This patient without apparent immunodeficiency presented with cryptococcal meningitis complicating communicating hydrocephalus similar to other reports [1, 2, 4, 6, 8]. However, this patient was initially misdiagnosed with normal pressure hydrocephalus requiring VP shunt placement, although the CSF culture completed 2 yrs earlier had revealed the presence of *C. gattii* infection. Chronic, undiagnosed *C. gattii* meningitis following the VP shunt placement lead to multiple recurrent communicating hydrocephalus, and eventually resulted in a cryptococcal intraabdominal VP shunt pseudocyst. A summary of nine case reports of cryptococcal VP shunt infection including the present case is provided in Table 1 [9–14]. Only three patients had immunodeficient conditions such as HIV infection (2) and diabetes mellitus with liver cirrhosis (1), while the six cases left had no apparent immunodeficient states. Previous studies tentatively found an association among human leucocyte antigen (HLA) subtypes and fungal infections such as histoplasmosis [15], paracoccidioidomycosis [16]. A study conducted in Papua New Guinea revealed patients conferred HLA B*5601 were likely susceptible to *C. gattii* infection [17]. However, a relation of HLA and genetic susceptibility to fungal infections is still inconclusive and requires more studies. In Asian population including Thai patients, anti-IFN-Ƴ autoantibodies have associated with disseminated infection secondary to several opportunistic organisms, such as nontuberculous mycobacteria, *Salmonella* non-Typhi, Varizella-zoster virus and fungi [18]. In addition, high level of anti-GM-CSF autoantibodies was associated with cryptococcal meningitis [19, 20] and disseminated cryptococcosis [21] in HIV-negative patients who had no previously apparent immunodeficiencies. The present case had negative results of the both autoantibodies. Unfortunately, information on the immunological studies of the previous cases in the Table 1 have been unknown. From the Table 1, cryptococcal VP shunt infection may be separated into early onset, within several months to a few years post implant, and late onset more than 10 years post implant. We postulate that an undiagnosed *Cryptococcus* infection might be the primary cause of neurological deficits in patients who presented with early onset infection. These patients received VP shunt placements to relieve their symptoms, but an occult *Cryptococcus* infection subsequently causes a shunt infection soon after. *Cryptococcus* can also cause a sporadic VP shunt infection in patients who had the shunt implant several years earlier. Of nine cases, three also had concurrent infected abdominal pseudocysts and one had a subcutaneous pseudocyst following the VP shunt infections. Clinical manifestations such as abdominal distention, abdominal pain, or abdominal mass were comparable among these patients. The infected pseudocysts were moderate to large in size, and cyst fluid tested for cryptococcal antigens from two patients showed high titers.

**Table 1:** Summary of case reports of cryptococcal meningitis complicating ventriculoperitoneal (VP) shunt infection with or without concurrent infected intraabdominal VP shunt pseudocyst

Case Author (year)	Age (yrs)/ Sex	Underlying conditions	Onset^a^ (mo-yrs)	Clinical manifestations	Findings	*Cryptococcus* species	Managements	Outcomes
1–3 Mangham et al. [9] (1983)	22/M	–	1 yr	Rapidly declined consciousness with frontal headache	CT brain: hydrocephalus Brain necropsy: basilar meningitis with yeast like organisms seen	*C. neoformans*	ABD, 5-FC, shunt removal	Dead
	58/M	–	9 mo	Progressive headache and memory deficit	CT brain: hydrocephalus Intact shunt function	*C. neoformans*	ABD, 5-FC, shunt removal	Recovery
	55/M	CLD, DM, NPH	4 mo	Memory deficit and gait difficulty	CT brain: hydrocephalus Brain necropsy: fibrous and thickening leptomeninges with yeast like organisms seen	*C. neoformans*	ABD, 5-FC	Dead
4 Crum-Cianflone et al. [10] (2008)	34/M	HIV, TB meningitis	1 yr	Abdominal distention	CT abdomen: intraabdominal CSF VP shunt pseudocyst, sized 26 cm Cyst fluid CRAG titers of 1:64	*C. neoformans*	LAB, 5-FC then oral FLU and 5-FC, cyst aspiration and shunt removal	Recovery
5 Viereck et al. [11] (2014)	65/M	NPH	20 yrs	Difficult ambulation and confusion	Intact shunt function Radiographic findings: no data	*C. neoformans*	ABD, 5-FC, shunt removal and reimplant	Recovery
6 Lee et al. [12] (2016)	80/M	NPH	10 yrs	Abdominal pain and diarrhea	CT abdomen: large intraperitoneal CSF VP shunt pseudocyst	*C. neoformans*	ABD, 5-FC and shunt removal	Recovery, no residual pseudocyst
7 Foong et al. [13] (2016)	52/M	NPH	1 yr	Fever, lethargy, confusion	CT brain: hydrocephalus with possible shunt malfunction	*C. neoformans*	LAB, 5-FC then oral FLU, shunt removal and reimplant	Recovery
8 Genebat et al. [14] (2017)	36/F	HIV, TB meningitis	1 yr	Abdominal mass	CT abdomen: subcutaneous CSF VP shunt pseudocyst, sized 7 cm	*C. neoformans*	LAB, 5-FC then oral FLU, and shunt removal	Recovery, no residual pseudocyst
9 The present case	66/F	HT	2 yrs	Gait difficulty, dizziness, headache, abdominal mass	CT brain: hydrocephalsCT abdomen: intraabdominal CSF VP shunt pseudocyst, sized 15 cm Cyst fluid CRAG titers of > 1:1024	*C. gattii*	ABD, FLU then oral FLU, shunt removal and reimplant	Recovery, no residual pseudocyst

^a^Temporal onset of infection following VP shunt implant

Abbreviations: *ABD* amphotericin B deoxycholate, *CLD* chronic liver disease, *cm* centimeter. *CRAG* cryptococcal antigen, *CSF*, cerebrospinal fluid, CT computed tomography, *DM* diabetes mellitus, *5-FC* 5-flucytosine, *F* female, *FLU* fluconazole, *HIV* human immunodeficiency virus, *HT* hypertension, *LAB* liposomal amphotericin B, *M* male, mo month, NPH normal pressure hydrocephalus, *TB* tuberculosis, yrs. years

The present case is unique for several reasons. First, this patient was initially misdiagnosed as NPH for 2 years. The reasons of slow progression in this patient may be from the released CSF by VP shunt at the onset of NPH, an ability to produce biofilm formation and a variation of virulence based on strain typing. Previous experimental studies elucidated *C. neoformans* exhibited exopolymeric matrices including capsular polysaccharide promoting adherence to VP shunt [22] and plastic surface [23], and was resistant to host immune response causing persistent infection. Second *C. gattii* was the only pathogen isolated from CSF and fluid from the pseudocyst in the present case while *C. neoformans* was the principle etiologic agent in the other cases. Third, intraabdominal VP shunt pseudocyst infection caused by *C. gattii* has not been previously reported*.* Molecular typing of the isolate identified *C. gattii* VGI strain, an uncommon type. According to the molecular typing studies, *C. gattii* VGII is the most common molecular type distributed in Thailand and other countries [3, 4]. Of 386 *Cryptococcus* isolates from Thailand, 12 (3.1%) were *C. gattii* VGII and only 1 (0.3%) was a VGI strain [3]. Interestingly, multi-locus sequence typing (MLST) revealed all but 1 of the *C. gattii* VGII had an identical linkage to the genotype of Vancouver outbreak strains [3]. Clinical manifestation from each molecular type were not significantly different, but most of the confirmed cases who died had an infection caused by the subtyped VGIIa (67%) and the subtyped VGIIb (27%) [4]. Several studies demonstrated that the subtyped VGIIa was more virulent than other strains [2, 24–26]. The present case had a CNS infection caused by the rare VGI strain. The insidious onset and slow progression of the CNS infection we observed might be associated with the less virulent VGI strain. The MICs of antifungal agents, such as ABD, 5-FC and azoles were active against the *C. gattii* isolated from our patient. However, the standard breakpoint of antifungal susceptibility testing for *Cryptococcus* spp. is still not available. Fluconazole resistance is uncommon among *C. gattii* although increasing fluconazole MICs of *C. gattii* VGII has been reported [27–29]. Thus, molecular typing and antifungal susceptibility testing may affect treatment outcomes in *C. gattii* infection.

Most of the case reports received a combination of conventional or liposomal amphotericin with 5-FC, which is the first line antifungal therapy for treating severe cryptococcal infection [7]. In the present case, high dose fluconazole was substituted because 5-FC is restricted use and not widely available in Thailand. Fluconazole is an alternative agent used for combination with amphotericin when treating HIV-infected patients with severe *C. neoformans* infection [7]. Although amphotericin B in combination with fluconazole showed in vitro antagonistic interaction [30], several clinical studies demonstrated the combined two-drug regimen provided favorable outcomes for treating HIV-infected patients with cryptococcal meningitis where 5-FC was not available or contraindicated [31–33] However, clinical studies of cryptococcal meningitis in non-HIV infected individuals have been limited. All cases listed in Table 1 that survived continued the oral fluconazole therapy for several weeks. Following the induction of combined antifungal agents, patients who have cryptococcal infection require long term fluconazole therapy to prevent relapse [7]. Neurological complications such as increased intracranial pressure or hydrocephalus, must be managed by CSF drainage procedures. The present case initially received a VP shunt to reduce hydrocephalus though the first CSF culture was positive for *C. gattii* at the onset. It is possible that placement of the VP shunt may have prevented a rapid deterioration in neurological status. Of the nine reported cases, eight had the VP shunt removal, and three of these including the present case subsequently had a shunt reimplant. All of these patients had neurological recovery and no recurrence following the reimplantation. Thus, in patients with cryptococcal meningitis complicating VP shunt infection, shunt removal followed by a reimplant is considered safe and provides a good outcome. Four patients who had concurrent infected abdominal pseudocysts required no surgical excision. Simple aspiration of the infected pseudocyst and prolonged antifungal therapy provided a favorable outcome.

## Conclusion

*C. gattii* infection should be considered in patients who develop normal pressure hydrocephalus without apparent cause. VP shunt implant is usually performed to relieve neurological deficits. An undiagnosed cryptococcal infection can result in VP shunt infection and infected intraabdominal VP shunt pseudocyst. CSF examination, cryptococcal antigen testing and fungal culture are mandatory to diagnosis this condition. Long term antifungal treatment, removal of the infected shunt followed by reimplantation when appropriate results in a favorable outcome.

## Abbreviations

5-FC: 5-flucytosine
ABD: Amphotericin B deoxycholate
anti-GM-CSF: Anti-granulocyte-macrophage colony-stimulating factor
anti-IFN-Ƴ: Anti-interferon gamma
BUN: Blood urea nitrogen
CGB: L-canavanine-glycine bromothymol blue
CNS: Central nervous system
CSF: Cerebrospinal fluid
CT: Computed tomography
HLA: Human leucocyte antigen
Ig: Immunoglobulin
MICs: Minimal inhibitory concentrations
NPH: Normal pressure hydrocephalus
RFLP: Restriction fragment length polymorphism
VP shunt: Ventriculoperitoneal shunt
WBC: White blood cell
